# Ten-year recurrence-free survival in stage IV perforated appendiceal mucinous adenocarcinoma with high-risk features: a case report

**DOI:** 10.1097/RC9.0000000000000770

**Published:** 2026-08-07

**Authors:** Yasushi Yamanaka, Machiko Kaizuka, Naoki Ishimaru, Tomoyuki Yamashita, Koji Asano

**Affiliations:** a Department of Surgery, Suwa Central Hospital, Chino, Nagano, Japan; b Department of Oncology, Suwa Central Hospital, Chino, Nagano, Japan; c Department of Pathology, Suwa Central Hospital, Chino, Nagano, Japan

**Keywords:** appendiceal mucinous adenocarcinoma, case report, cytoreductive surgery, pseudomyxoma peritonei, recurrence-free survival, systemic chemotherapy

## Abstract

**Introduction and importance:**

Appendiceal mucinous adenocarcinoma (MAC) associated with pseudomyxoma peritonei (PMP) has a poor prognosis in advanced stages. Cytoreductive surgery with hyperthermic intraperitoneal chemotherapy (CRS/HIPEC) is generally regarded as the standard treatment for PMP, but its management may require individualization based on tumor burden, histopathological risk, the patient’s condition, and available treatment options.

**Presentation of the case:**

A 71-year-old man was initially managed conservatively for suspected acute appendicitis and was later referred to our department because of persistent appendiceal enlargement. Before the planned outpatient evaluation, he developed perforated appendicitis and underwent emergency laparoscopic appendectomy. Pathological examination showed MAC with high-risk features and a positive surgical margin. After multidisciplinary discussion, additional surgery aimed at macroscopic disease reduction was performed without CRS/HIPEC, including right hemicolectomy, lymph node dissection, and resection of visible peritoneal nodules and a paracaval lymph node. Postoperative FDG-PET/CT suggested residual pelvic disease, and systemic chemotherapy with capecitabine, oxaliplatin, and bevacizumab was administered. A clinical complete response was achieved, and the patient has remained recurrence-free for 10 years.

**Clinical discussion:**

This case suggests that long-term recurrence-free survival may be possible when the biologically active tumor burden is limited, timely surgery achieves substantial macroscopic disease reduction, and effective systemic chemotherapy controls residual disease, even if CRS/HIPEC is not performed.

**Conclusion:**

Long-term RFS may be achievable in carefully selected patients with stage IV appendiceal MAC and PMP, even without CRS/HIPEC. This case does not challenge CRS/HIPEC as the standard strategy for PMP but supports individualized multidisciplinary decision-making when CRS/HIPEC is not feasible or not selected.

## Introduction

Appendiceal mucinous adenocarcinoma (MAC) associated with pseudomyxoma peritonei (PMP) is a rare malignancy with a poor prognosis in advanced stages^[^1–3^]^. Prognosis is influenced by several high-risk features, including high-grade histology, signet-ring cell components, lymph node metastasis, perforation-associated peritoneal disease, and the extent of residual disease^[^1–9^]^. Cytoreductive surgery with hyperthermic intraperitoneal chemotherapy (CRS/HIPEC) is generally regarded as the standard treatment for PMP from appendiceal tumors^[^10–13^]^. However, recent consensus statements and clinical practice guidelines also recognize that treatment selection must be individualized according to tumor biology, disease distribution, patient condition, and the feasibility of complete cytoreduction^[^10,12,13^]^. This report describes a patient with stage IV MAC and high-risk features who achieved long-term recurrence-free survival (RFS) without CRS/HIPEC. We focus on treatment decision-making and factors that may explain this unusual outcome. This case report is reported in accordance with the SCARE checklist^[^14^]^.

HIGHLIGHTS

- Ten-year recurrence-free survival was observed in a case of stage IV appendiceal mucinous adenocarcinoma with pseudomyxoma peritonei.
- High-risk features included perforation, positive margin, nodal disease, peritoneal involvement, and nonregional lymph node metastasis.
- Macroscopic disease reduction, followed by systemic chemotherapy, achieved a clinical complete response without cytoreductive surgery with hyperthermic intraperitoneal chemotherapy (CRS/HIPEC).
- This case supports individualized, multidisciplinary treatment when standard CRS/HIPEC is not feasible.

## Presentation of the case

A 71-year-old man presented to a local clinic with right lower-quadrant abdominal pain. Acute appendicitis was suspected on clinical grounds, and because symptoms improved with oral antibiotics, conservative management was selected. He had no known family history of appendiceal or colorectal cancer. After symptom resolution, follow-up computed tomography (CT) was performed at the referring clinic because of the atypical clinical course in an elderly patient and to exclude an underlying appendiceal lesion. CT demonstrated persistent appendiceal enlargement. He was therefore referred to our hospital.

On presentation to our department, he was asymptomatic. Serum carcinoembryonic antigen and carbohydrate antigen (CA) 19-9 were normal. Colonoscopy showed no cecal tumor, and biopsy specimens showed no malignancy.

**Figure 1.**
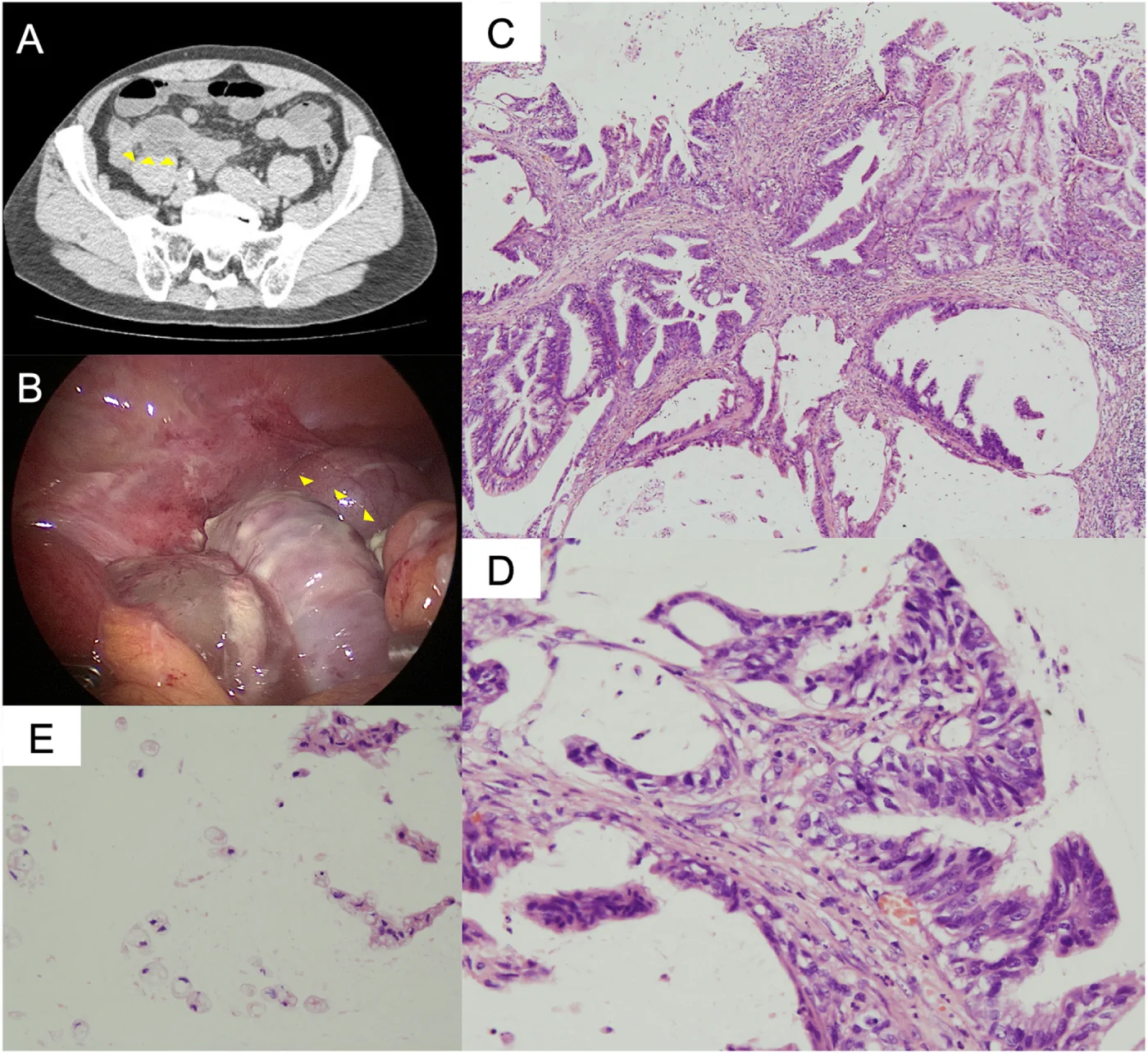
Findings at appendiceal perforation and pathology of the primary tumor. (A) Noncontrast CT image at the time of appendiceal perforation demonstrated fat stranding around the appendix and a periappendiceal fluid collection (arrows). (B) Intraoperative findings during the initial surgery showed that the appendiceal wall was necrotic and perforated in the midportion (arrows), with abscess formation and mucin pooling in the pelvic cavity and bilateral paracolic gutters. (C) Hematoxylin and eosin (H&E) stain (×40) of the primary tumor. The tumor was a mucinous adenocarcinoma (MAC), and invasion into the mesoappendix was confirmed. (D) H&E stain (×200). Deep invasion of MAC into the subserosa was confirmed. (E) Pathological findings show tumor cells floating in the mucin pool. Although initially diagnosed as true signet ring cells, these cells were ultimately reclassified as pseudo-signet ring cells because they were floating in the mucin pool without invasive growth.

On Day 13, he developed recurrent abdominal pain late at night and presented to our emergency department on Day 14. An emergency non-contrast CT scan was performed because physical findings suggested peritonitis. CT revealed a swollen appendix, periappendiceal fat stranding, ascites, and suspected abscess formation (Fig. 1A). A diagnosis of perforated appendicitis with peritonitis was made, and an emergency laparoscopic appendectomy was performed.

Intraoperatively, an abscess cavity extended into the pelvic space and both paracolic gutters. The appendix was inflamed and perforated at the necrotic midportion, with mucin pooling in the abscess cavity (Fig. 1B).

**Figure 2.**
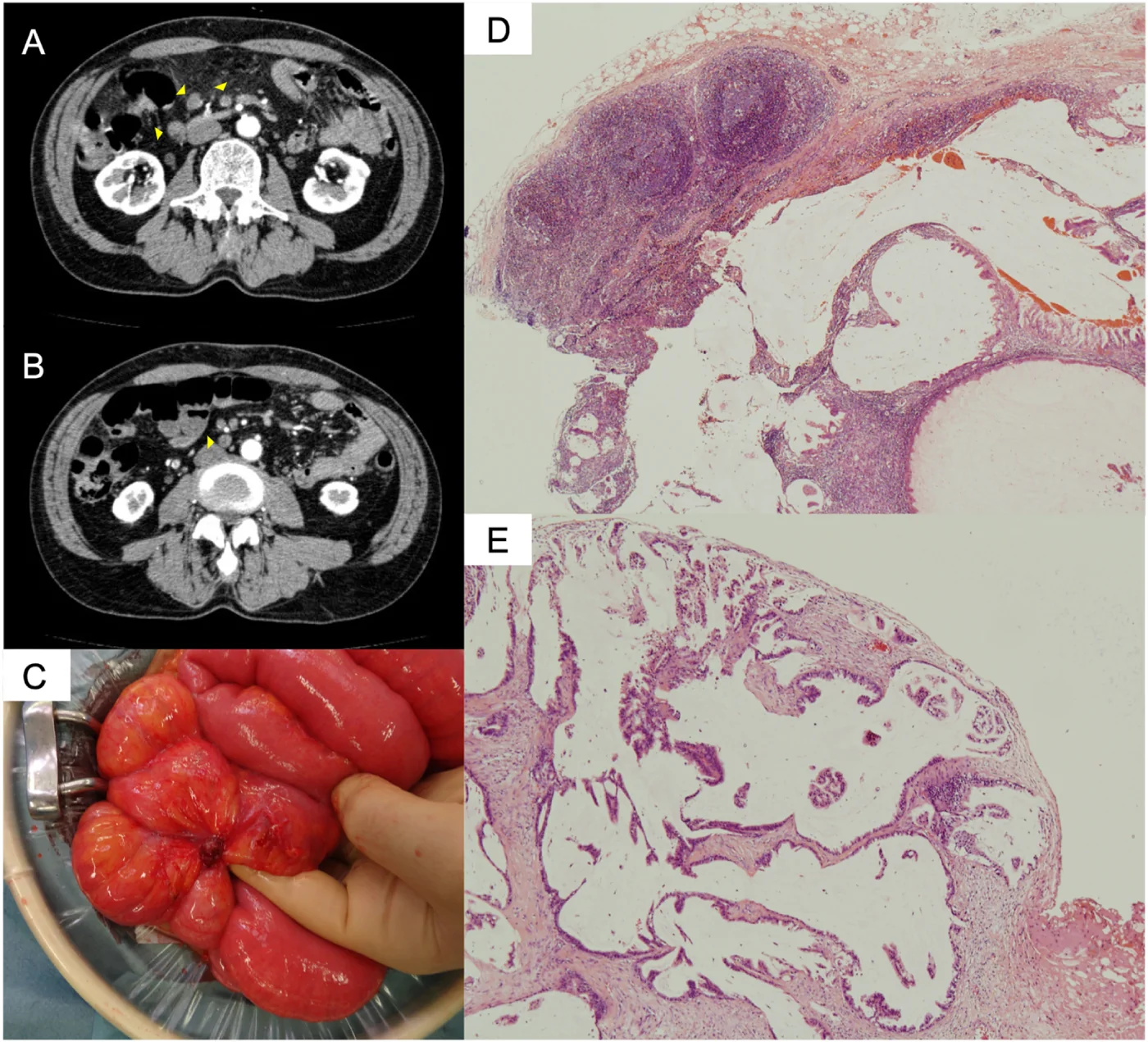
Staging and pathological confirmation of lymph node and peritoneal involvement. (A) Contrast-enhanced CT image before the second surgery. Multiple enlarged lymph nodes (LNs) were noted in the ileocolic artery (ICA) and right colic artery (RCA) regions (arrows). (B) Contrast-enhanced CT image before the second surgery. Paracaval LN enlargement was observed (arrow). (C) Intraoperative findings during the second surgery. Peritoneal nodules were scattered mainly in the small-bowel mesentery, including the pelvic space. All 10 macroscopically visible nodules were resected. (D) H&E stain (×40) of the paracaval LN. Metastasis of MAC was confirmed. (E) H&E stain (×40) of a resected peritoneal nodule. Metastasis of MAC was confirmed within the nodule.

On Day 19, pathological examination confirmed MAC. Because of the positive margin, perforation-associated mucinous disease, and suspected nodal involvement, several treatment strategies were considered, including referral to a CRS/HIPEC center, systemic chemotherapy alone, and additional macroscopic disease reduction followed by systemic chemotherapy. On Day 20, these options were explained to the patient, who preferred to continue treatment at our hospital. After a multidisciplinary team discussion involving surgery, medical oncology, and pathology, and shared decision-making with the patient, additional macroscopic disease reduction followed by systemic chemotherapy was selected. Preoperative laboratory tests showed elevated CA19-9 (93.0 U/mL) and CA 125 (108.8 U/mL) levels. Contrast-enhanced CT for staging demonstrated multiple enlarged ileocolic and right colic lymph nodes and an enlarged paracaval lymph node (Fig. 2A and B). No ascites or distant organ metastasis was observed.

On Day 48, right hemicolectomy with D3 lymph node dissection was performed while preserving the middle colic artery, and was followed by functional end-to-end anastomosis. The paracaval lymph node was resected. Several small nodules measuring 5–13 mm and suspicious for peritoneal dissemination were identified in the small-bowel mesentery and resected (Fig. 2C).

The surgical margin was negative. Metastatic mucinous adenocarcinoma was found in 6 of 12 regional lymph nodes (pN2) and in a paracaval nonregional lymph node (pM1c) (Fig. 2D). One of the 10 resected mesenteric nodules showed metastatic mucinous adenocarcinoma, while the remaining nine contained only mucin deposition (Fig. 2E). The final diagnosis was stage IVC MAC with signet ring cell components, Grade G3.

**Figure 3.**
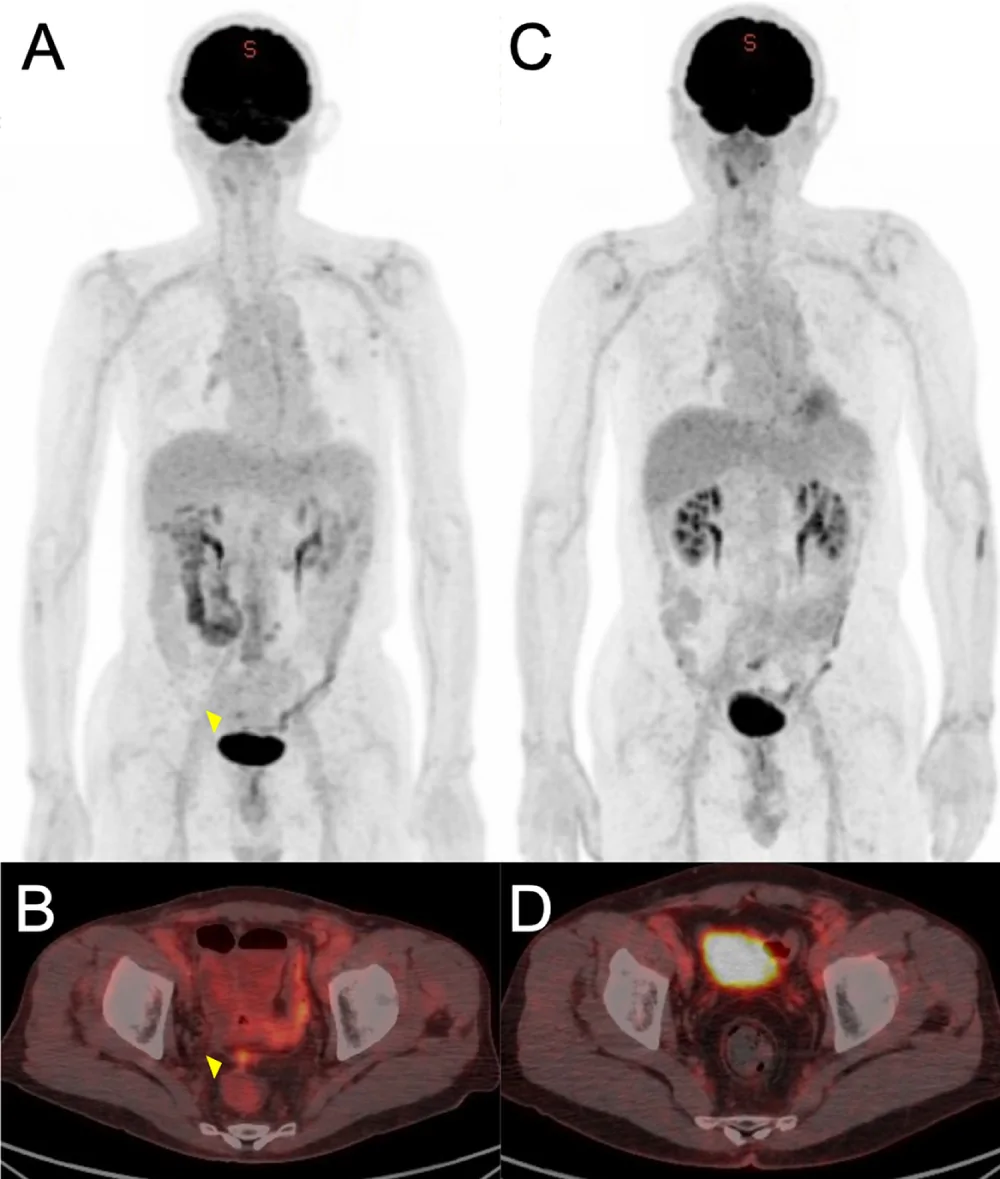
Post-chemotherapy fluorodeoxyglucose (FDG) PET/CT findings compatible with a clinical complete response. (A) Axial PET image before chemotherapy showing abnormal FDG uptake in the pelvic cavity (arrow). (B) Coronal PET/CT image before chemotherapy demonstrating increased uptake in the pelvic cavity [maximum standardized uptake value (SUVmax) of 4.0] (arrow). (C) Axial PET image after completion of chemotherapy showing resolution of FDG uptake in the previously noted lesion. (D) Coronal PET/CT image after completion of chemotherapy demonstrating complete resolution of pelvic FDG uptake, compatible with a clinical complete response.

On Day 91, fluorodeoxyglucose positron emission tomography/computed tomography (FDG-PET/CT) demonstrated residual FDG uptake in the rectovesical pouch (SUVmax 4.0; Fig. 3A and B), and systemic chemotherapy was planned. The first course of capecitabine plus oxaliplatin (XELOX) plus bevacizumab was initiated on Day 105. On Day 120, a strangulated small-bowel obstruction occurred after the first course and before the second course, requiring emergency adhesiolysis. On Day 140, the second course of XELOX was given without bevacizumab, which was resumed on Day 154. A total of 17 cycles of XELOX-based chemotherapy were administered over 13 months. Post-treatment FDG-PET/CT showed complete resolution of the residual FDG uptake, with no new peritoneal dissemination or distant metastasis (Fig. 3C and D). Serum CA19-9 and CA125 levels returned to normal. The patient was judged to have achieved a clinical complete response. From Year 2 onward, CT showed no recurrence. He remained recurrence-free at the most recent 10-year follow-up. The clinical course and treatment sequence are summarized in Table 1.

**Table 1:** Clinical timeline.

Time point	Clinical event
Day 0	Acute appendicitis was suspected, and oral antibiotics were started at a local clinic.
Day 11	Persistent appendiceal enlargement was noted on follow-up CT, and elective outpatient evaluation at our hospital was scheduled.
Day 13	Recurrent abdominal pain developed late at night.
Day 14	Emergency CT suggested perforated appendicitis, and laparoscopic appendectomy was performed.
Day 19	Pathology confirmed appendiceal mucinous adenocarcinoma.
Day 20	Treatment options, including CRS/HIPEC referral, were explained; the patient preferred treatment at our hospital.
Day 48	Additional cytoreductive surgery was performed.
Day 91	PET/CT suggested residual disease, and systemic chemotherapy was planned.
Day 105	XELOX plus bevacizumab was initiated.
Day 120	Strangulated small bowel obstruction required emergency adhesiolysis.
Day 140	The second course of XELOX was administered without bevacizumab.
Day 154	XELOX plus bevacizumab was resumed.
Month 13	XELOX-based chemotherapy was completed, with complete resolution of residual FDG uptake.
Year 2 onward	Surveillance showed no recurrence.
Year 10	The patient remained recurrence-free.

## Discussion

This case report describes a patient with stage IV MAC with regional nodal metastases (pN2), paracaval nonregional lymph node metastasis (pM1c), and perforation-associated PMP. The notable feature is durable 10-year RFS after staged macroscopic disease reduction and systemic chemotherapy without CRS/HIPEC. At the time of initial treatment, the Peritoneal Surface Oncology Group International/European Reference Network for Rare Adult Solid Cancers (PSOGI/EURACAN) clinical practice guidelines and the Chicago Consensus on peritoneal surface malignancies had not yet been published^[^10,12^]^. Current guidance supports CRS/HIPEC as the standard approach for PMP, while also emphasizing case-by-case decision-making according to tumor biology, disease extent, patient condition, and cytoreduction feasibility^[^10,12,13^]^.

The first relevant factor was pathological risk stratification. In appendiceal tumors, histopathological evaluation is essential for standardized reporting and prognostic assessment^[^3–5^]^. The initial pathology report described mucinous adenocarcinoma with signet-ring cell components and assigned grade G3. Subsequent pathological re-review showed that the signet ring–appearing cells were confined to mucin pools without stromal invasion, consistent with pseudo-signet ring cells^[^3,6–9^]^. This reinterpretation did not remove the high-risk nature of the case, given the positive margin, PMP, regional nodal metastases, and nonregional nodal metastasis^[^6,10^]^. However, it may partly explain why durable disease control was possible despite the initially unfavorable histological impression.

The second factor was the apparent discordance between macroscopic disease extent and biologically active tumor burden. Extra-appendiceal mucin extended beyond the right lower quadrant, supporting classification as PMP rather than as localized extra-appendiceal mucin^[^1^]^. The peritoneal nodule containing neoplastic epithelium showed destructive invasion, consistent with high-grade mucinous carcinoma peritonei (PMCA), which is generally associated with a poorer prognosis than disseminated peritoneal adenomucinosis (DPAM) but a better prognosis than PMCA with signet-ring cells^[^1,2^]^. Nevertheless, only one of 10 resected nodules contained neoplastic epithelium, whereas the remaining nodules contained acellular mucin. This predominance of acellular mucin suggested that the biologically active tumor burden was limited relative to the visible disease burden^[^1,3,13^]^.

The third factor was the sequencing of treatment. CRS/HIPEC remains the standard treatment strategy for PMP arising from appendiceal tumors^[^10–13^]^. Because CRS/HIPEC was not institutionally available at that time, referral to a CRS/HIPEC center was discussed with the patient as an alternative. However, after consideration of disease distribution and the patient’s preferences following an explanation of the available options, treatment at our hospital was selected. Although the staged operation was not a formal CRS/HIPEC procedure, right hemicolectomy, regional lymph node dissection, paracaval lymph node resection, and resection of all visible peritoneal nodules were completed within 1 month after perforation. This surgical component may have reduced the macroscopic disease burden before extensive cellular peritoneal disease became established.

The fourth factor was systemic chemotherapy. Systemic chemotherapy for MAC with PMP has generally been based on regimens used for colorectal cancer. Current recommendations support fluoropyrimidine-based chemotherapy combined with oxaliplatin, with consideration of an anti-angiogenic agent in selected settings^[^6,10^]^. In this case, postoperative FDG-PET/CT raised concern for residual pelvic disease, and capecitabine plus oxaliplatin combined with bevacizumab was initiated. PMCA generally shows limited sensitivity to systemic chemotherapy^[^2,13^]^. However, Liu *et al* reported meaningful disease control with capecitabine, oxaliplatin, and bevacizumab in mucinous appendiceal adenocarcinoma, particularly when cellular peritoneal lesions or lymph node metastases were present^[^15^]^. The normalization of tumor markers and resolution of FDG uptake support the possibility that systemic therapy contributed to control of residual cellular disease.

This case should therefore be interpreted cautiously. The favorable outcome does not imply that surgery without HIPEC should be considered equivalent to CRS/HIPEC in stage IV MAC with PMP. The course might have differed if the patient had more extensive cellular peritoneal disease, incomplete macroscopic disease reduction, rapidly progressive tumor biology, or an inadequate response to chemotherapy. Because current guidelines do not specify surveillance beyond 5 years, further follow-up will be individualized at extended intervals^[^16^]^.

## Conclusion

Long-term RFS may be achievable in carefully selected patients with stage IV MAC and PMP, even without CRS/HIPEC, when biologically active tumor burden is limited, timely surgery achieves a substantial macroscopic disease reduction, and systemic chemotherapy effectively controls residual disease. While this case does not challenge CRS/HIPEC as the standard strategy for PMP, it supports individualized multidisciplinary decision-making when CRS/HIPEC is not feasible or not selected.

## Data Availability

The data that support the findings of this study are available from the corresponding author upon reasonable request.
